# Hydrogels Based on Polyacrylamide and Pectin Containing Rice Husk Ash: Preparation, Characterization and Application in Formulation of Cementitious Materials

**DOI:** 10.3390/ma17235746

**Published:** 2024-11-24

**Authors:** Ruth Hevellen Sousa Rodrigues, Edson Araujo de Almeida, Fábio Rodrigo Kruger, Edson Cavalcanti Silva-Filho, Edvani Curti Muniz

**Affiliations:** 1 Chemistry Postgraduation Program, Federal University of Piaui—UFPI, Teresina 64049-550, PI, Brazil; 2 Chemistry Postgraduation Program, State University of Maringa—UEM, Maringá 87020-900, PR, Brazil; 3 Civil Construction Department, Federal University of Technology-Parana—UTFPR-CM, Campo Mourão 87301-889, PR, Brazil; 4 Materials Science Postgraduation Program, Federal University of Technology-Parana—UTFPR-LD, Londrina 86036-370, PR, Brazil

**Keywords:** hydrogel, mortar, mechanical resistance, rice husk ash

## Abstract

Superabsorbent polymers (PSAs) have been extensively studied to act as internal curing agents in cementitious materials, as they have the characteristic of absorbing and releasing water in a controlled manner, which can contribute to the hydration process of a cementitious medium during its consolidation. Thus, hydrogels consisting of polyacrylamide (PAAm), pectin (Pec) and rice husk ash (RHA) were synthesized to be applied in cementitious matrices. In addition, the PSAs were characterized by FTIR, SEM, and XRD. For evaluating the usage of hydrogels as internal curing agents, different hydrogel contents—0.03, 0.06, and 0.1 (wt-%, relative to cementitious components)—were used for mortar preparation. The mechanical strengths of the cementitious materials were evaluated at day 7 and day 28 during the curing process. The addition of PSAs to the mortars caused an increase in mechanical resistance such that the 0.06% content presented better performance at day 7 of curing (4.07% higher) and at day 28 of curing (8.06% higher) when compared with the reference mortar (without the addition of PSAs) in the same curing periods. This work demonstrates that the addition of PSAs contributes to the hydration of a cementitious material, improving the mechanical resistance of the studied mortars.

## 1. Introduction

In recent years, there has been a great advance in technologies aimed at civil construction [1,2]. In this context, concrete and mortar are among the cementitious materials used the most in the structural part of civil construction [1] due to their positive characteristics, such as resistance, durability, moulding, accessibility, and the abundance of raw materials [2,3].

The durability of cementitious composites depends on several factors, such as the conditions that the material will be exposed to during its useful life (e.g., environmental action), and on factors related to its formulation, components, and structures, such as the type of cement, water/cement ratio [4,5], and additives to minimize crack formation [5]. The occurrence of cracks in a structure at an early age caused by shrinkage problems can also compromise the strength and durability of cementitious materials [5]. Shrinkage in cementitious materials occurs due to excessive water loss and a decrease in its resistance [6].

Among several methods which have been widely used to minimize the effect of shrinkage and simultaneously guarantee the resistance of cementitious materials, the application of so-called curing agents [6,7] can be highlighted. This technology is based on a basic procedure in which adequate humidity and temperatures are provided to the cementitious material to promote hydration of the material for a certain period [8]. Proper curing is crucial to achieving strength and therefore greater durability, especially for cementitious materials exposed to extreme environmental conditions at an early age [9]. This method can be divided into two groups: external curing and internal curing.

Internal curing occurs with a supply of water to the existing aggregates inside the cementitious material [7,10]. For this, water penetrates the cementitious material through its capillaries. Thus, the supply of water minimizes and compensates for water evaporation and increases the degree of hydration [11]. The internal curing of the cementitious compound can also occur through the addition of a curing agent in the formulation which will serve as an internal water reservoir. Such an agent can continuously and gradually release water as hydration occurs [7,12].

Several types of materials can be used as internal curing agents, among them being superabsorbent polymers (PSAs), rice husk ash (RHA), pre-moistened lightweight aggregates (LWAs), and fly ash (FA) [13,14]. Due to their characteristics, PSAs have become a very promising type of material for use as an internal curing agent, mainly due to their ability to absorb a significant amount of liquid, if immersed in aqueous environments and retain the liquid within its structure without dissolving [15]. The liquid absorbed by the PSAs can be released continuously and gradually over time to the outside (i.e., to the environment constituted by the elements of the cementitious material) [5]. It has been demonstrated that the use of PSAs can contribute to the hydration reaction of the material and minimize the shrinkage process through more adequate internal curing [16,17].

The use of an SAP as an internal curing agent is a subject which has been etensively studied due to its water release and desorption kinetics, which are directly related to the physical and chemical properties of the PSA [18,19]. In the literature, there are studies showing that the addition of an SAP improves the hydration of the medium, being able to prevent self-desiccation and mitigate autogenous shrinkage [19,20]. However, there is no consensus on how an SAP influences the mechanical property, as some studies showed that the addition of SAPs causes a reduction in mechanical strength [21,22], while others reported an increase in mechanical strength [23,24].

One hypothesis for the reduction in mechanical strength may be related to the particle size of the PSA incorporated into the cementitious medium [20,25], because the larger the SAP particle, the greater the amount of water absorbed or released, which can improve the hydration of the medium but can also lead to the formation of large pores, which can negatively affect the resistance of the material [19].

Some PSAs can absorb amounts of water up to 5000 times their own weight, in addition to having a three-dimensional network structure [15]. They are characterized by having a strong affinity for water due to the presence of hydrophilic groups, such as -OH, -COOH, -CONH_2_, and -SO_3_H, in their structure [17].

As for their classification, PSAs (hydrogels) can be classified as natural or synthetic, or they can be obtained from a combination of natural and synthetic polymers. PSAs have a high degree of flexibility due to their high water content [26,27,28]. Currently, the incorporation of natural polymers in the preparation of hydrogels for application in the areas of health and agriculture, for example, has been widely used to improve applicability, biocompatibility, and biodegradability [29]. Pectin hydrogels are often used [30,31] due to pectin being from natural sources. Pectin (Pec) is a polysaccharide of plant origin consisting of poly α 1-4-galacturonic acids found in the cell walls of terrestrial plants. It has a linear structure, is non-toxic and biocompatible, and has gelling, mucoadhesive, thickening, and emulsifying properties [32,33,34,35,36]. Due to these properties, this polysaccharide is widely used for the development of hydrogels [34]. Acrylamide is a vinylamide (CH_2_=CH-CONH_2_)—a white solid soluble in water—and is widely used for the synthesis of polyacrylamide through a polymerization reaction [37,38]. Rice husk (RH) is a byproduct obtained through the processing of rice. Its chemical composition consists of an organic part (74%) which includes cellulose, hemicellulose, and lignin and an inorganic part (26%) composed mainly of silicon dioxide (SiO_2_) and other constituents, such as silicon oxide (SiO_2_), alumina (Al_2_O_3_) sulphur trioxide (SO_3_), iron oxide (Fe_2_O_3_), calcium oxide (Ca_2_O), magnesium oxide (MgO), sodium oxide (Na_2_O), and potassium oxide (K_2_O). RH is considered a biomass, and therefore it is widely used for energy generation through its burning, which leads to the production of a residue called rice husk ash (RHA]) [39,40].

Considering the addition of a PSA as an internal curing agent, another advantage, aside from the release of additional water to make up for water lost through evaporation, is that it is also possible to control the rheological properties [14]. However, after the release of water by a PSA, formation of empty micropores in the hardened material may occur, thus increasing the porosity of the material and reducing its mechanical properties [14,30]. One way to minimize this problem is to incorporate materials such as rice husk ash (RHA), which after the release of water by a PSA can partially fill the pores formed and thus improve the mechanical resistance of the material.

RHA is a material that has a high silica (SiO_2_) content, which makes it a pozzolanic material that, when added to the cementitious medium, can contribute to increasing the mechanical resistance. In addition, RHA is an agricultural byproduct, and seeking new applications for this byproduct contributes to reducing environmental problems caused by the incorrect disposal of this material [41,42,43].

The hypothesis evaluated in this work is that the incorporation of RHA would increase the mechanical properties in the hydrogel of PAAm, pectin, and RHA. In this work, hydrogels based on polyacrylamide (PAAm), pectin (Pec), and rice husk ash (RHA) are produced, in which the amounts of these components vary in different formulations through a 2^3^ full factorial design in triplicate at the central point. The hydrogels are incorporated into formulations of cementitious materials (mortar) and characterized through measurements of the mechanical properties in periods of 7 and 28 days after preparation.

## 2. Materials and Methods

### 2.1. Materials

The hydrogels were prepared using apple pectin (CAS 900-69-5, Sigma-Aldrich, Saint Louis, MO, USA) with a degree of methoxylation of 50–75%; acrylamide (Fisher Scientific, USA); potassium persulfate (Synth, Diadema, Brazil); N,N’-methylenebisacrylamide 99.5% (CAS 110-26-9, Fisher Scientific, Waltham, MA, USA); and rice husk, which was purchased from the municipality of Boqueirão do Piauí, PI, Brazil. Portland cement CP II-F and sand (Brazilian normal in 4 fractions: coarse (maximum size: 1.19 mm); medium coarse (maximum size: 0.59 mm); medium fine (maximum size: 0.297 mm), and fine (maximum size: 0.149 mm)) were used to produce the mortar according to ABNT/NBR 7214/2015 [44].

### 2.2. Methods

#### 2.2.1. Preparation of RHA and Synthesis of the Hydrogels

The rice husk ash (RHA) was obtained through calcination in a muffle furnace at 600 °C for 2 h and cooled to room temperature inside the muffle for 24 h. After this procedure, the ash produced was ground and sieved through a 325 mesh (44 µm) sieve [45].

A 2^3^ full factorial design in triplicate at the central point was used to produce hydrogels. The Q value was used to analyze the influence of three factors: the acrylamide (AAm) content, Pec content, and RHA content. The levels of each factor were adjusted based on preliminary studies, and the values for the upper (+), middle (0), and lower (−) levels are shown in Table 1. The levels of the three factors were combined, resulting in 11 experiments, as described in Table 2, with experiments 9a, 9b, and 9c being replicates of the central point.

For the hydrolysis of the hydrogels, 40 mL of 0.5 mol L^−1^ NaOH solution was used per 1 g of dry hydrogel. In a hood, the solution was magnetically stirred for 1 h at 50 °C. Then, the hydrogels were washed with distilled water until reaching a pH of 7 and subsequently dried in an oven at 60 °C for 24 h [41]. In the preparation of hydrogels in each of the different formulations (different experimental conditions), the levels of the factors described in Table 1 and their combinations, as described in Table 2, were used. For the synthesis of hydrogels, the pectin was first dissolved in distilled water (necessary volume to maintain the total concentration at 136 g L^−1^) at 50 °C. The solution was then kept at room temperature and stirred for 24 h. After pectin solubilization, rice husk ash was added to obtain a suspension. For this, the system was kept under agitation (by means of a magnetic stirrer) at 70 °C. Then, the required amount of acrylamide (AAm), 0.04 g of N,N’-methylenebisacrylamide (MBAAm), and 0.08 g of sodium persulfate were added, with an interval of 10 min between the addition of each reagent in the sequence.

The hydrogels obtained were divided into small pieces and left immersed in distilled water to be washed (removal of reagents not incorporated into the hydrogel). The water was renewed every 1 h for a total period of 6 h. The obtained material was then dried in an oven at 60 °C for 24 h [31]. In addition, the hydrogels which were applied to the cementitious compound, after drying, were crushed and sieved until obtaining the granulometry of a 250 mesh.

#### 2.2.2. Preparation of Mortars and Cement Pastes

Four formulations of mortars and cement pastes were prepared with a water/cement ratio of 0.48 according to NBR 7215 (ABNT/1996) [46] and traces at a ratio of 1:3 (cement: sand). The hydrogel used was H4 (formulation 4; see Table 2), with 0.03%, 0.06%, and 0.1% hydrogel in relation to the mass of the cement. A reference formulation (Ref) without the addition of hydrogel was also implemented following the same procedure, and it was used for comparison in the characterization tests. The as-prepared hydrogels were left to swell for 20 h in tap water before being used to prepare the cementitious material. The fine aggregate was normal sand inserted at 4 different granulometries: fine sand, medium-fine sand, medium-coarse sand, and coarse sand.

Cement pastes were prepared by mixing water and cement with and without the addition of hydrogel. The pastes containing 0.03%, 0.06%, and 0.1% hydrogel were labeled P-0.03, P-0.06, and P-0.1, respectively, and the one without hydrogel was labeled P-Ref.

To prepare the mortar, the mixer was first turned on, and fine aggregates and cement were added to the tank. The mixture was maintained at a low speed for 60 s, and then water was added with the hydrogel swollen until equilibrium. Then, the material was maintained at a high speed for 60 s. After this time, the mixer was turned off, and the material which remained on the sides of the vat was removed with a spatula and placed inside the vat. Then, the materials were mixed for another 2 min at high speed.

After preparing the mortar, the test specimens were moulded into a cylindrical shape 50 mm in diameter and 100 mm in height. They were prepared and labeled as Mort0.03, Mort0.06, Mort0.01, and Mort-Ref. After a period of 24 h, the samples were cured at room temperature (25 °C) and with a relative humidity of 72%.

### 2.3. Characterization

#### 2.3.1. Fourier Transform Infrared Spectroscopy (FTIR)

The FTIR spectra of the hydrogels, RHA, and cement paste were obtained in a Bruker Vertex 70 spectrometer in the range of 400–4000 cm^−1^, with a resolution of 4 cm^−1^. Powdered samples were prepared using the pelleting technique in 1% KBr (64 scans).

#### 2.3.2. X-Ray Diffraction (XRD)

Samples of rice husk ash, cement paste, and hydrogels were characterized by X-ray diffraction in a Shimadzu XRD-600 (Kyoto, Japan) using a CuKα radiation source, with a voltage of 40 kV and current of 30 mA. Data were collected at a speed of 1° min^−1^, whose sweep range was (2θ) from 3 to 75°.

#### 2.3.3. Scanning Electron Microscopy (SEM)

The morphologies of the hydrogels were analyzed using a scanning electron microscope (FEI, model Quanta 250, Pittsburgh, PA, USA). To obtain the images, the samples were covered with a thin layer of gold of approximately 50 nm. The images were obtained with an accelerating voltage of 20 kV. The average pore diameters of the hydrogels were analyzed using Image-J^®^ 1.8.0 software by manually delimiting the pore contours observed in the SEM images.

#### 2.3.4. Thermogravimetric (TG and DTG)

The samples of the cement pastes were characterized in a Shimadzu DTG-60 to analyze the hydration process of the pastes. The tests were carried out at room temperature initially and up to a final temperature of 600 °C, with a heating rate of 10 °C and a N_2_ flow at 100 mL min^−1^.

#### 2.3.5. Water Swelling, Desorption Test, and Testing of the Drying and Swelling Effects of Hydrogels

Swelling tests were used to study the water absorption of the hydrogels immersed in different media, including distilled water, a buffer solution (pH: 4, 7, and 9), and a saline solution (NaCl). All solutions (buffer and saline) had a constant ionic strength of 0.1 mol L^−1^. In this way, dry hydrogels with a mass of approximately 0.06–0.1 g were immersed in a beaker with 30 mL of a solution or distilled water. The hydrogels were removed at different time periods (every 1 h for a total period of 8 h at 24 h, 48 h, and 72 h). In each case, excess water from the sample was removed, and then the hydrogel was weighed on an analytical balance. For each hydrogel formulation (Table 2), three trials were performed (*n* = 3). The swelling capacity (measured with the Q parameter) could be calculated according to Equation (1), where m_1_ is the mass of the dry material and m_2_ is the mass of the swollen hydrogel:

Q = [(m_2_ − m_1_)/m_1_] (1)

For the desorption assay, the hydrogels swollen to equilibrium (m_2_) were placed in an oven at 30 °C. The samples were weighed at different drying time intervals. For each hydrogel formulation (Table 2), tests were performed in triplicate (*n* = 3).

The process of the repeat effect of drying and swelling of the hydrogels was verified in an aqueous medium. The dried hydrogel was immersed in water, and the absorption was determined as previously described above. The material was then dried at 60 °C for 24 h and swollen again. The process was then repeated 5 times.

#### 2.3.6. Resistance Test of Cementitious Materials

The compressive strength of the mortar was measured following the recommendations of NBR 7215 (ABNT/1996) [46] at ages of 7 and 28 days, using an EMIC Pc 100 testing machine (mechanical press). For the compression test, the test specimen was placed in the center of the lower plate of the mechanical press, and then the equipment was turned on, exerting force until the test specimen ruptured.

For each formulation, 4 mortar test specimens were used, testing each prepared mix (*n* = 4). The compressive strength result was obtained by averaging the individual resistance values of each test specimen of the same age through the quotient between the force recorded by the equipment and the area of the cross-section of the test specimen where the force was applied.

## 3. Results

### 3.1. Kinetic Behavior of Water Absorption by Hydrogels

**Figure 1:**
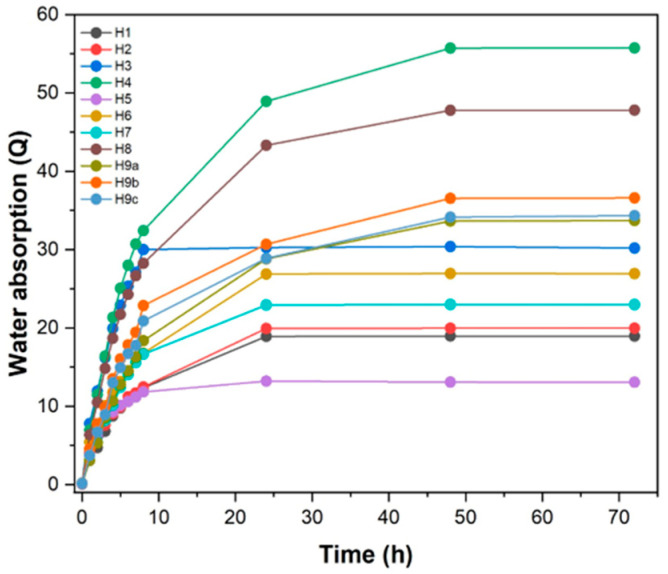
Absorption of hydrogels in water.

Figure 1 shows the degree of swelling (Q) of the hydrogels in distilled water for different immersion times. For all samples, the degree of swelling (Q) increased rapidly in the first few hours. It can be observed that both the H3 and H5 hydrogel formulations reached the equilibrium value after 8 h of immersion, while the other hydrogel formulations reached approximately 90% of the equilibrium in 24 h. Thus, the H3 and H5 formulations showed quicker swelling processes and reached equilibrium faster. In addition, the other hydrogel formulations showed a slower capacity to absorb water at equilibrium. The swelling degrees of H4 and H8 (Q, in g/g) were 55.72 and 47.77, respectively, which were higher compared with the other hydrogels. Therefore, it is inferred that the Q values and the time required to reach swelling equilibrium depend on the formulation of each hydrogel [47]. Thus, it can be said that the H4 hydrogel presented better water absorption due to the higher starch and pectin contents in its composition, which means that the number of hydrophilic groups was greater, thus favoring water absorption.

It can also be observed from Table 2 that the H4 and H8 hydrogels had similar formulations, differing only in the amount of RHA, which emphasizes that the H8 formulation hydrogel had a higher amount of RHA. This explains the reduced swelling of the hydrogel of the H8 formulation compared with that of the H4 formulation. Such a reduction in the Q value may be related to the presence of a greater amount of RHA which filled the hydrogel pores and, at the same time, reduced the elasticity of the matrix [45], aspects which led to a decrease in the degree of swelling.

Since the H4, H8, and H9 hydrogels showed higher degrees of swelling if compared with the other hydrogels prepared in this work, these were chosen to carry out the subsequent analyses with the objective of evaluating which was the best hydrogel to be applied in cementitious materials.

### 3.2. Kinetic Behavior of Water Absorption of Hydrogels at Different pH Levels and in Saline Solution

**Figure 2:**
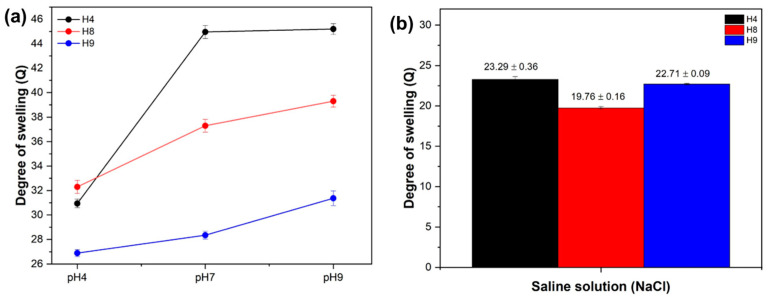
(**a**) Degree of swelling of hydrogels at different pH levels and (**b**) in saline solution.

Figure 2a shows the dependencies of the degree of swelling of hydrogels H4, H8, and H9 at different pH values, and the dependence of the degree of swelling on the saline solution is shown in Figure 2b. It can be seen in Figure 2a that at a pH of four, the H9 hydrogel had a lower Q value than the H4 and H8 hydrogel formulations. Furthermore, at a pH of four, the three hydrogels showed lower water absorption values, and as the pH of the solution increased from 4 to 7 and from 7 to 9, subsequent increases in the Q value occurred, which may be explained as follows. In an acid medium (pH of four or less), the carboxyl groups are mostly in protonated form (-COOH), and thus there is little (or no) electrostatic repulsion between them [48,49]. It is emphasized that the pKa of carboxylic groups is close to 4.0–4.5 [31,50]. Thus, at pH levels of seven and nine, the carboxylic groups were in dissociated form (-COO^−^). Thus, at pHs of seven and nine (pH > pKa), the carboxylate anions presented electrostatic repulsion among themselves. This induced a greater inflow of water (or aqueous liquids) into the hydrogel matrix to increase the distance between the negatively charged groups and thus reduce electrostatic repulsion, leading to a greater degree of swelling. Thus, the hydrogel matrix consequently swelled more and expanded [31,45].

The influence of the salinity of the solution on the degree of swelling of the hydrogels (in 0.1 mol L^−1^ of NaCl solution) is shown in Figure 2b. Tests in a saline environment were performed for evaluating the influence of salts on the swelling degree of the hydrogels through the factor f in Equation (2). A value close to 1.0 for f indicates a weaker effect of salts on the swelling properties of the hydrogel. Therefore, the H4 hydrogel was the best while H9 was the worst when the hydrogels were swollen in 1 mol L^−1^ aqueous NaCl. The swelling capacity of the hydrogels in the saline solution decreased in relation to the swelling in distilled water (Figure 1) and the buffer (Figure 2b), and the degree of swelling of samples H4, H8, and H9 in saline at its retention equilibrium was about 23.296 ± 0.36, 19.767 ± 0.16, and 22.713 ± 0.09, respectively. It can be explained that this reduction was due to the sodium cations (Na^+^) interacting in the saline solution, with the pectin carboxylate groups forming ionic complexes, which provided a decrease in the electrostatic repulsion in the polymeric matrix [51]. With the decrease in electrostatic repulsion, less water would be required to minimize repulsion, and therefore less water needed to diffuse into the hydrogel, and the degree of swelling would be lower. In addition, the sensitivity factor (ƒ) of the hydrogels to salt is defined by Equation (2) [52,53]:

ƒ = 1 − [Qsal/Qwater] (2)

where Qsaline is the degree of swelling in the saline solution and Qwater is that in the distilled water. The ƒ values of the hydrogels (Table 3) show that the H9 hydrogel had a lower saline effect compared with the H4 and H8 hydrogels, which had similar saline effects. This is an indication that the ash content did not affect the value of ƒ.

### 3.3. Desorption of Swelling Water from Hydrogels

**Figure 3:**
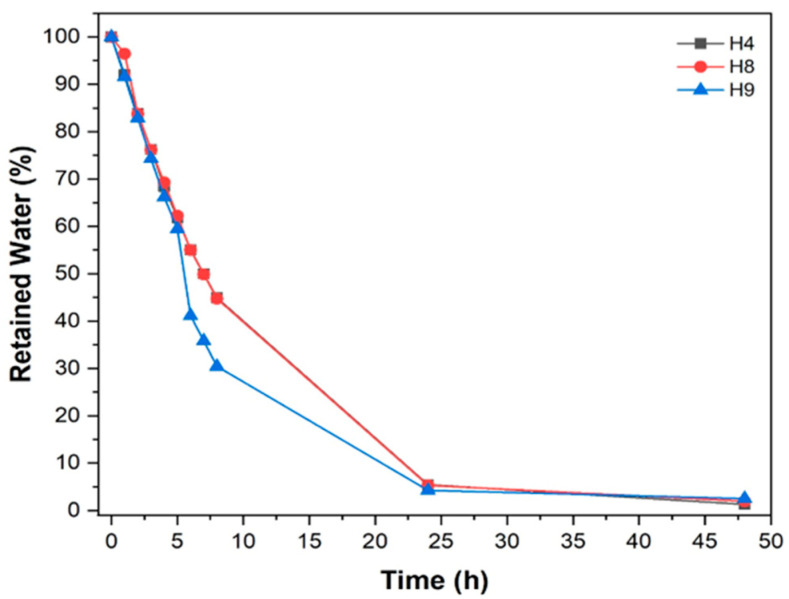
Release profiles of adsorbed water in hydrogels H4, H8, and H9.

The release kinetics of the water adsorbed by hydrogels H4, H8 and H9, are shown in Figure 3. Each point in this figure represents an average obtained from the triplicates (*n* = 3). The water release from the H4 and H8 hydrogel formulations was approximately equal over time (up to 48 h). Therefore, the points almost completely overlapped. It can be observed that about 50% of the water adsorbed by the H4 and H8 hydrogels was released in the first 7 h of the experiment, while the H9 hydrogel in the same period released approximately 35% of the total swelling water contained therein. Furthermore, about 95% of all of the water contained in the hydrogel matrices was released within 24 h. In 48 h, the three hydrogels almost completely lost the adsorbed water, but it is known that it is quite difficult for the hydrogel to lose 100% of the water contained in its matrix [17]. It can be observed that the H4 and H8 hydrogels presented rather similar water release profiles throughout the experiment time (0–48 h). The formulations to obtain these hydrogels differed only in their RHA content, with the H4 hydrogel having an RHA content factor at a lower level and that of H8 being at an upper level (Table 2).

### 3.4. Testing of the Drying and Swelling Effects of Hydrogels

The water’s release profiles were measured for evaluating the time in which the water molecules diffused out of the hydrogel when it was maintained in an oven at a controlled temperature. When inside the cementitious material, the time for the water to diffuse out of hydrogels would be higher. Therefore, it was expected that this could be related to the time in which the water acted as a curing agent inside the cementitious material. In the H4 and H8 hydrogel formulations, the AAm and Pec content factors were maintained at their upper levels. Thus, it appears that the RHA content did not influence the kinetics of water release. Knowledge of the water release profile by hydrogels is a highly important aspect for obtaining an adequate application of the hydrogel as a curing agent in cementitious materials. As was already mentioned, the continuously and gradually released water would contribute to cement hydration and thus improve the internal curing process.

**Figure 4:**
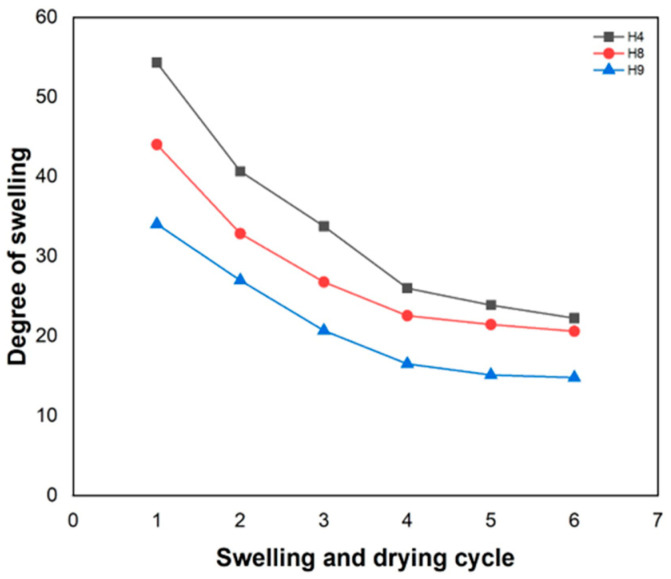
Effect of swelling and drying cycles of hydrogels on water absorption capacity.

Figure 4 shows the effect of repeating the swelling and drying cycle of the hydrogels on the degree of swelling. It was observed that all samples showed an initial decrease in the swelling degree value (Q) and only stabilized after the fourth drying and swelling cycle. When the hydrogel met water molecules, it swelled and could expand to many times its initial volume. Thus, repetition of the drying and swelling cycle affected the water absorption capacity [45]. The H4 hydrogel showed greater reduction (59%) in the Q value after the fifth swelling and drying cycle, while the H8 and H9 hydrogels showed a reduction of 53% and 56%, respectively.

### 3.5. FTIR, XRD, and Morphology Analysis of Hydrogels

**Figure 5:**
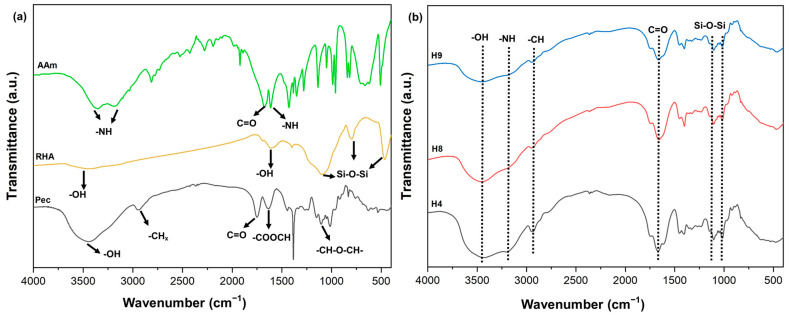
FTIR spectra of (**a**) Pec, RHA, and Aam and (**b**) H4, H8, and H9 hydrogel formulations.

The FTIR spectra of AAm, RHA, and Pec are shown in Figure 5a. In the FTIR spectrum of the RHA bands at 1091, 786 and 466 cm^−1^, symmetric and asymmetric elongations and angular deformations of the Si-O-Si bonds of the silica present in the material were observed [45]. The bands at 3492 and 1614 cm^−1^ can be attributed to the O-H stretching and deformation vibrations of the silanol group, respectively [45,46,47,48,49,50,51,52,53,54]. The AAm FTIR spectrum showed characteristic bands at 3377 and 3195 cm^−1^, which can be attributed to asymmetric and symmetric elongation of the amide (-NH) groups. The bands at 1674 and 1604 cm^−1^ corresponded to C=O elongation and the N-H bond [55]. In the pectin spectrum, bands at 3464 and 2951 cm^−1^ were observed, which refer to the stretching vibration of -OH and -CHx, respectively.

The bands at 1751 cm^−1^ and 1635 cm^−1^ can be attributed to the elongation vibrations of the C=O groups present in the -COOCH group. In addition, the band at 1022 cm^−1^ was due to –CH–O–CH– elongation [33,56,57]. In addition to the bands already mentioned for PAAm, RHA, and Pec, it was observed that the spectra of hydrogels H4, H8, and H9 (Figure 5b) were similar, showing bands at 3456 cm^−1^ and 3207 cm^−1^ which corresponded to the -OH and -NH stretching vibrations. The band at 2937 cm^−1^ refers to the C-H stretching vibration, and the band at 1666 cm^−1^ refers to the C=O stretching vibrations of the amide group of PAAm [58]. There was also the presence of a band at 466 cm^−1^, attributed to the Si-O-Si binding of RHA. Therefore, it can be concluded that the appearance of these bands showed that the hydrogels containing PAAm, pectin, and rice husk ash (RHA) were successfully synthesized.

**Figure 6:**
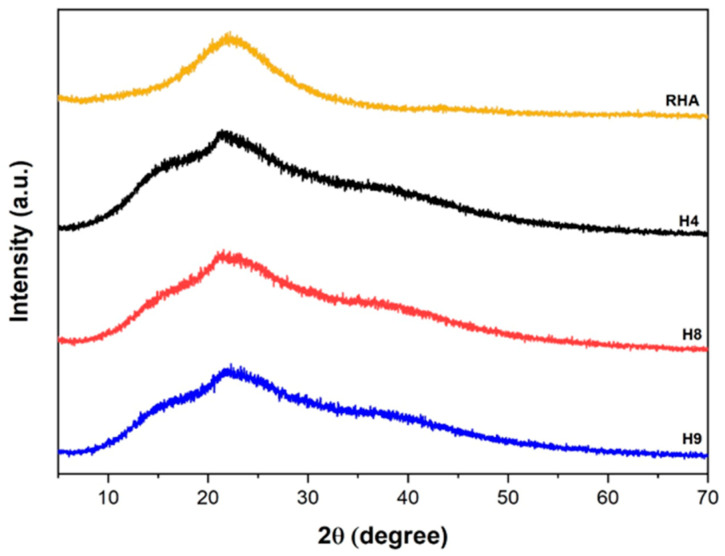
XRD diffractograms of RHA and H4, H8, and H9 hydrogel formulations.

Figure 6 presents XRD diffractograms of pristine rice husk ash and hydrogels H4, H8, and H9. The diffraction pattern shows that the ash diffraction pattern had no defined diffraction peaks; only a broad peak at 2θ = 22.3° is displayed, showing the non-crystalline phase of silica [29,45]. The diffractogram of the hydrogels showed an amorphous structure which is characteristic of a disorganized system and the presence of a peak referring to the amorphous silica of the RHA. Thus, it can be concluded that the RHA was incorporated into the polymer matrix of the hydrogel during the gelation process.

**Figure 7:**
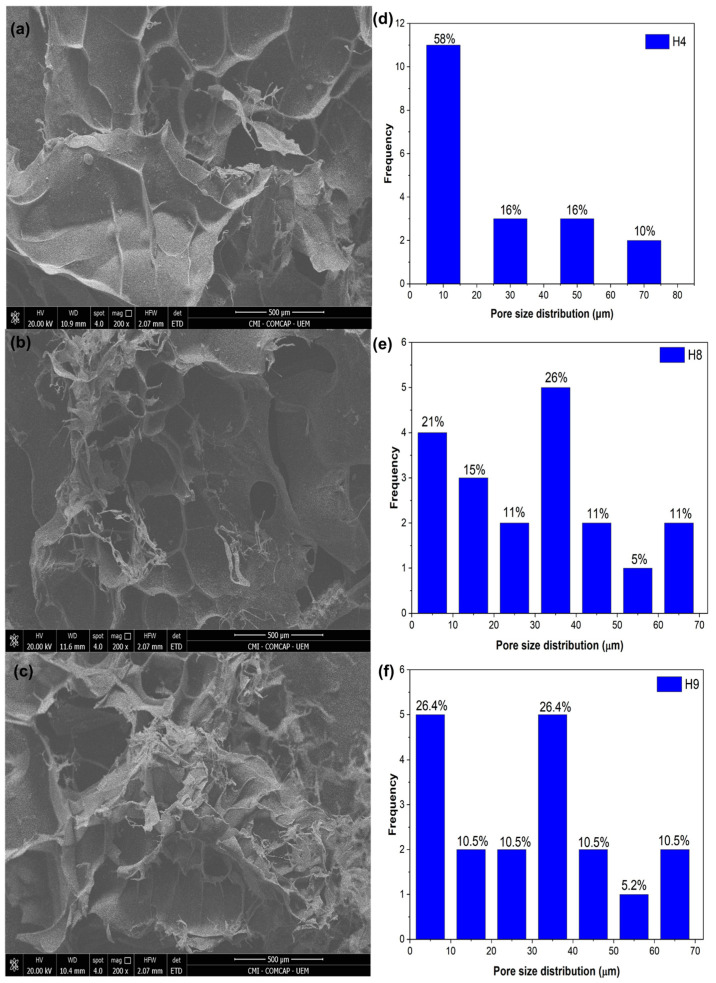
SEM micrographs of the hydrogels: (**a**) H4, (**b**) H8, and (**c**) H9. Magnification: 200×. (**d**–**f**) Pore size distribution.

SEM images of the H4, H8, and H9 hydrogels are shown in Figure 7a–c. All hydrogels exhibited porous structures with irregular shapes, in addition to pores of different sizes which were distributed throughout the hydrogel matrices. The distributions of the average size for 19 pores (*n* = 19) of the hydrogels, which were calculated through their areas, are shown in Figure 7d–f. The average pore sizes of the H4, H8, and H9 hydrogels were 20.82, 30.13, and 29.75 µm, respectively. It was observed that H8 showed a larger average pore size in relation to the other two hydrogel formulations. This indicates that the introduction of RHA into preparation of the hydrogels increased the size of the pores, making a structure with pores larger than those of the others.

### 3.6. FTIR, XRD, and TG/DTG Analysis of Cement Pastes

**Figure 8:**
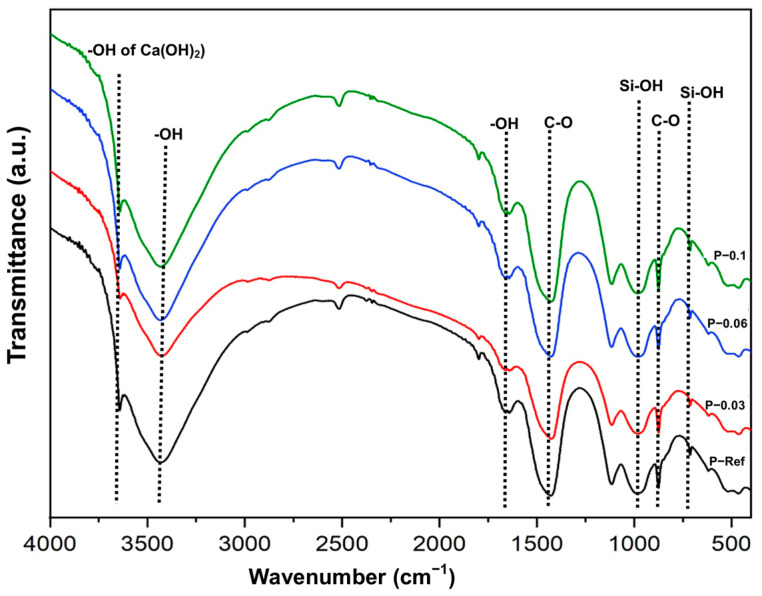
FTIR of cement pastes at day 7.

Figure 8 presents the FTIR spectra of the cement pastes after 7 days of curing. The cement pastes containing hydrogels presented FTIR spectra quite similar to the reference paste. The FTIR spectra of the cement pastes had bands at 3460 and 1672 cm^−1^, which can be attributed to symmetric and asymmetric elongation related to vibration and bending of the O-H groups of water molecules [59,60]. The band observed at 3649 cm^−1^ is characteristic of calcium hydroxide (Ca(OH)_2_) due to the stretching vibration of the O-H bond [59,61]. The bands at 1456 and 873 cm^−1^ are related to elongation vibrations of the C-O bonds in calcium carbonate [62,63]. The spectra of the pastes had bands at 979 and 711 cm^−1^, which can be attributed to bending vibrations of the Si-OH bonds in C_2_S and C_3_S [59,62,63]. According to the analyses carried out in the FTIR spectra, it was possible to identify the hydrated compounds present in the cement samples. Furthermore, the modified cement pastes containing different hydrogel contents contained the same hydrated compounds as the reference paste.

**Figure 9:**
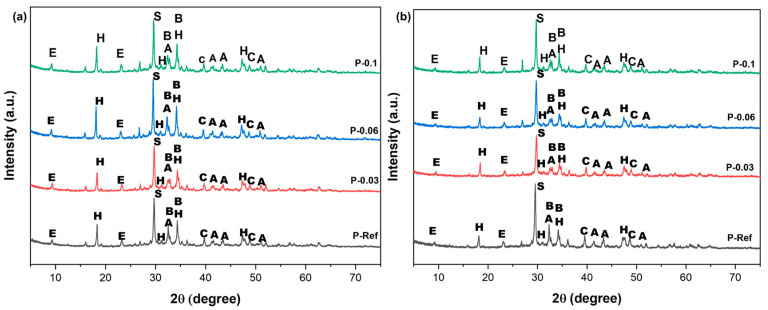
XRD of cement pastes at 7 days and 28 days. (**a**) Paste aged 7 days. (**b**) Folder aged 28 days. Note: calcium hydroxide (H), ettringite (E), hydrated calcium silicate (S), belite (B), alite (A), and calcite (C).

The hydrogel chosen to be applied to the cementitious compounds was H4, based on the factorial design and because it presented better results in the swelling tests and exhibited a smaller pore size. After water desorption, the PSAs left empty pores in the hardened cementitious material. Thus, the smaller the pore size of the PSAs, the smaller the voids left by them. In Figure 9, the XRD profiles of the cement pastes at days 7 and 28 of curing are presented. The hydrated compounds identified in all cement pastes were calcium hydroxide (Ca(OH)_2_), calcite (CaCO_3_), ettringite, hydrated calcium silicate (C-S-H), belite (C_2_S), and alite (C_3_S). The diffractograms obtained for the reference pastes (without the addition of hydrogels) were like those of the pastes containing the hydrogels over time (at days 7 and 28 of curing), and this result shows that the use of different hydrogel contents in the formulation of the mortar did not alter the crystallographic structure of the hydrated cementitious material. However, it can be observed that the Ca(OH)_2_ peak increased with the addition of different hydrogel contents in the paste.

Considering the XRD profiles of the pastes which were analyzed at days 7 and 28 of curing, both were structurally quite similar. However, for the intensities of the peak of calcium hydroxide in the region of 2θ equal to 18.2 and 34.0°, a reduction occurred in the period of 28 days of curing when compared with the intensity of the same peak in the XRD profile of the paste at day 7 of curing. This was due to the pozzolanic reaction consuming Ca(OH)_2_ during the cement hydration process due to the presence of silica [12,64,65]. Through the diffractograms of the pastes, the main peaks which were found for calcium hydroxide at 2θ = 18.2, 31.8, 34.2, and 47.3°, hydrated calcium silicate at 2θ = 29.6°, ettringite at 2θ = 9.4 and 23.1°, belite at 2θ = 32.3 and 34.2°, alite at 2θ = 32.3, 41.1, 43.3, and 51.1°, and calcite at 2θ = 39.6 and 48.9° were responsible for the durability of the cementitious compound [64,66,67,68].

**Figure 10:**
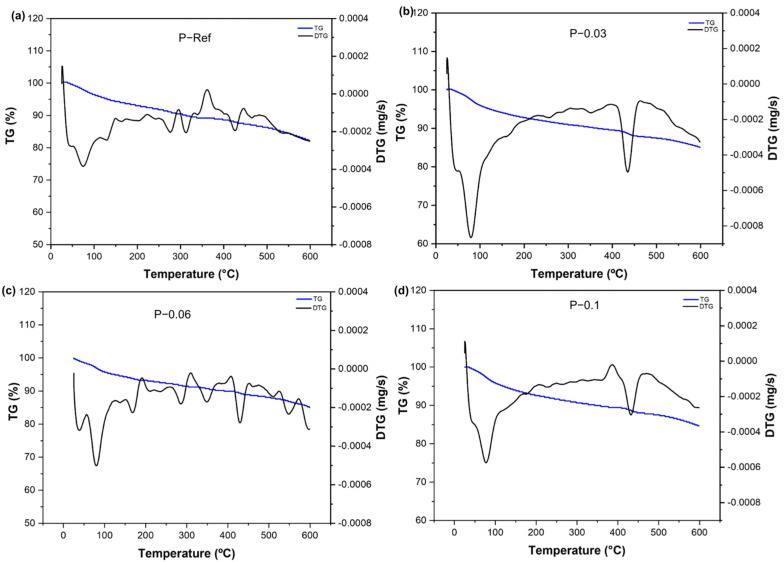
TG and DTG curves of cement pastes: (**a**) P−Ref, (**b**) P−0.03, (**c**) P−0.06, and (**d**) P−0.1.

Figure 10 shows the TG and DTG curves of the cement pastes containing hydrogels and the references in which they were analyzed after 90 days of curing. It can be observed that all of the pastes presented mass loss at temperatures of 25–105 °C, which corresponds to evaporation of the absorbed water [69,70]. Furthermore, it was observed that the TG curves of the hydrogel-containing pastes were like the reference paste, indicating that no new events were found. The DTG curves of the pastes showed peaks at 78 °C associated with the release of water in the pores and the dehydration of the hydrated compounds, namely hydrated calcium silicate and ettringite [69,71,72], having a peak with a maximum at 430 °C corresponding to the dehydration of the Ca(OH)_2_ [69,70].

### 3.7. Compressive Strength of Cement Mortar

**Figure 11:**
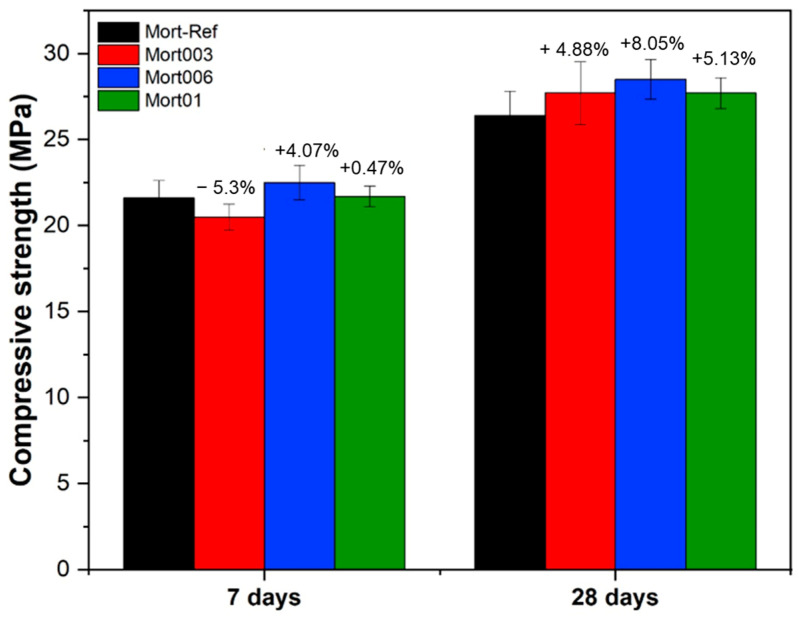
Compressive strength of cement mortars containing different hydrogel contents for aging at days 7 and 28.

The results for the compressive strength of cement mortars with the addition of different hydrogel contents (0.03%, 0.06%, and 0.1%) are shown in Figure 11. It can be seen that in the 7 day curing period, sample Mort006 showed the greatest increase in mechanical strength (an increase of 4.07%) and Mort01 showed a slight increase (an increase of 0.47%) in compressive strength compared with the reference mortar (Mort-Ref) in the same period. However, in sample Mort003, there was a 5.3% decrease in strength compared with the reference mortar.

It can be observed in Figure 11 that in the 28 day curing period, the compressive strengths of Mort003, Mort006, and Mort 01 increased by 4.88%, 8.05%, and 5.13%, respectively, in relation to the mechanical strength of Mort-Ref. In addition, it can be noted that in the Mort003 sample, there was a significant increase with 28 days of curing in relation to the resistance at 7 days. This may occur because the hydrogel releases water in a controlled manner, favoring the curing process of the mortar [31]. In addition, the reduction in strength may also be related to the voids in the mortar structure created by the hydrogels [20].

Through the results obtained in the resistance tests, it was possible to conclude that the addition of different hydrogel contents in the mortar did affect its resistance, since all of the samples showed an increase at day 28 of curing when compared with the reference one. In addition, the Mort006 sample was the one that showed the greatest increase in resistance over time in relation to the other grades studied.

To better analyze whether the hydrogel content and the age of the samples interfered with the compressive strength results, analysis of variance (ANOVA) was carried out at a confidence level of 95%, and the results are presented in Table 4, where it should be noted that the age and hydrogel content were statistically significant.

Therefore, based on the results obtained in this work, it can be observed that the incorporation of hydrogels based on PAAm, Pec, and RHA in cementitious materials (mortar) significantly improved the compressive strength. This increase may be associated with a good retention capacity and the subsequent release of stored water at the right time for the hydration process, considering the reactions which occurred during the setting time [73,74], thus contributing to the hydration reaction of the cementitious material. As expected, the influence of the age of curing affected the compressive strength more than the hydrogel.

## 4. Conclusions

Hydrogels based on polyacrylamide and pectin and containing rice husk ash were successfully synthesized, as can be seen from the FTIR, XRD, and SEM images. The hydrogel obtained from the H4 formulation showed a better Q value. In addition, the H4 and H8 samples showed a controlled and quite similar release of water over time. This makes these hydrogels rather interesting materials to apply to cementitious material as an internal curing agent, as it releases water at the right time for the setting process and thus contributes to cement hydration and improving its mechanical strength.

In this work, the influence of the addition of different contents of hydrogel formulation H4 for application in cement mortar at days 7 and 28 of curing was also studied. The result showed that the addition of hydrogels improved the mechanical resistance of the mortar, and the addition of 0.06% presented a better result for resistance to compression at day 7 of curing (4.07% higher) and at 28 days of curing (8.06% higher) when compared with the reference mortar (without the addition of PSAs). Furthermore, the FTIR, DRX, and TG analyses showed that the addition of the hydrogels in the pastes did not change the crystallographic structure or the chemical characteristics of the cement.

To the best of our knowledge, this is the first time that hydrogel formulations using pectin, rice husk ash (RHA), and crosslinked polyacrylamide were used as curing agents for cementitious materials. Beyond the technological use of hydrogel as a curing agent, this methodology is also interesting because it uses a natural product (pectin) and RHA as byproduct from rice agro-industries. Therefore, it should be also seen as a contribution to the environment. In this way, it can be concluded that the production of polyacrylamide hydrogels containing RHA and pectin can be used as an internal curing agent for cementitious media, in addition to adding commercial value to rice husk and preventing it from being discarded incorrectly and thus causing environmental problems.

## Figures and Tables

**Table 1:** Factors and respective levels used in factorial planning.

Factors (g)	Lower Level (−)	Central Point (0)	Upper Level (+)
AAm	1.5	2.5	3.5
Pec	1.0	2.0	3.0
RHA	0.1	0.2	0.3

**Table 2:** Factorial design matrix used for the synthesis of hydrogels.

Run	AAm Content	Pec Content	RHA Content	H_2_O (mL)
H1	−1	−1	−1	19.12
H2	+1	−1	−1	33.82
H3	−1	+1	−1	33.82
H4	+1	+1	−1	48.3
H5	−1	−1	+1	20.59
H6	+1	−1	+1	35.29
H7	−1	+1	+1	35.29
H8	+1	+1	+1	50.00
H9a	0	0	0	34.55
H9b	0	0	0	34.55
H9c	0	0	0	34.55

**Table 3:** The ionic strength function for H4, H8, and H9 hydrogels.

Hydrogel	H_2_O (g)	NaCl (1 mol L^−1^) (g)	ƒ
H4	55.723	23.296	0.581
H8	47.777	19.767	0.587
H9	36.535	22.713	0.371

**Table 4:** Analysis of variance of the compressive strength test data of the mortars, considering the main factors of age and the hydrolyzed hydrogel content.

Effect	SQ *	DF **	MQ ***	F	*p* Value	Result
Age	1.99 × 10^14^	11	1.99 × 10^14^	141.5	1.27 × 10^−7^	Significant
Hydrogel	2.00 × 10^14^	6	4.00 × 10^13^	18.2	5.30 × 10^−6^	Significant
Residue	1.55 × 10^13^	-	1.41 × 10^12^	-	-	-

* SQ = quadratic sum. ** DF = degrees of freedom. *** MQ = mean of squares.

## Data Availability

The original contributions presented in the study are included in the article, further inquiries can be directed to the corresponding author.
